# [6]-Gingerol Prevents Disassembly of Cell Junctions and Activities of MMPs in Invasive Human Pancreas Cancer Cells through ERK/NF-****κ****B/Snail Signal Transduction Pathway

**DOI:** 10.1155/2013/761852

**Published:** 2013-09-24

**Authors:** Sung Ok Kim, Mi Ryeo Kim

**Affiliations:** Department of Herbal Pharmacology, College of Oriental Medicine, Daegu Haany University, 165 Sang Dong, Suseong gu, Daegu 706-828, Republic of Korea

## Abstract

To study the effects of [6]-gingerol, a ginger phytochemical, on tight junction (TJ) molecules, we investigated TJ tightening and signal transduction pathways in human pancreatic duct cell-derived cancer cell line PANC-1. The following methods were utilized: MTT assay to determine cytotoxicity; zymography to examine matrix metalloproteinase (MMP) activities; transepithelial electrical resistance (TER) and paracellular flux for TJ measurement; RT-PCR and immunoblotting for proteins related to TJ and invasion; and EMSA for NF-**κ**B activity in PANC-1 cells. Results revealed that TER significantly increased and claudin 4 and MMP-9 decreased compared to those of the control. TJ protein levels, including zonula occludens (ZO-) 1, occludin, and E-cadherin, increased in [6]-gingerol-treated cells, which correlated with a decrease in paracellular flux and MMP activity. Furthermore, NF-**κ**B/Snail nuclear translocation was suppressed via downregulation of the extracellular signal-regulated kinase (ERK) pathway in response to [6]-gingerol treatment. Moreover, treatment with U0126, an ERK inhibitor, completely blocked NF-**κ**B activity. In conclusion, these findings demonstrate that [6]-gingerol regulates TJ-related proteins and suppresses invasion and metastasis through NF-**κ**B/Snail inhibition via inhibition of the ERK pathway. Therefore, [6]-gingerol may suppress the invasive activity of PANC-1 cells.

## 1. Introduction

Most natural products target multiple gene products and thus are ideally suited for the prevention and treatment of various chronic diseases, including cancer [1]. Metastasis is considered the major cause of death in patients with cancer. Recently, in various human cancers, including pancreatic cancer, some tight junction (TJ) proteins, including claudins, were determined to be abnormally regulated; thus, they may be promising molecular targets for diagnosis and therapy [2, 3].

TJs are apical intercellular junctional complexes whose general function is the maintenance of epithelial polarity, as well as functioning as selective barriers to molecules such as inhibition of solute and water flow through the paracellular space [4]. TJs are dynamic structures subject to modulation during wound repair, inflammation, and tumor progression. TJs are dysregulated or lost in cancer tissues. Consequently, dysregulation of TJ proteins contributes to cancer progression and metastasis [5]. 

**Figure 1:**
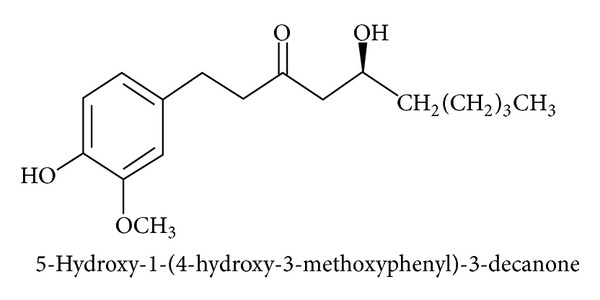
Chemical structures of [6]-gingerol.

The rhizome of *Zingiber officinale*, commonly known as ginger, is a globally important spice. The ginger phytochemicals, specifically [6]-gingerol, (5-Hydroxy-1-(4-hydroxy-3-methoxyphenyl)-3-decanone; Figure 1), the major pungent component of ginger, has antioxidant, anti-inflammation, and antitumor promoting activities [6–8]. However, the exact mechanism responsible for the anti-invasiveness and metastasis effects of ginger in pancreatic cancer cells is still unknown. The present research attempts for the first time to address TJ regulation in the anti-invasive effects of ginger in pancreatic cancer (PC) chemotherapy.

PC is one of the most lethal malignant cancers in Western countries, as well as in Korea [6, 7]. PC is characterized by rapid progression, late clinical presentation, difficulty in early diagnosis, and unresponsiveness to chemotherapy, radiotherapy, and immunotherapy, resulting in low resectability rates after diagnosis, early recurrence after resection, and extremely poor survival rates [8, 9]. At the time of diagnosis, PC normally shows extensive local invasion and/or metastasis, precluding a curative surgical resection. A better understanding of the molecular genetics of pancreatic carcinoma is needed to develop new diagnostic and therapeutic strategies. However, no studies have reported the effect of [6]-gingerol on the cellular and molecular mechanisms of invasion and metastasis related TJs in human pancreatic cancer cells. In this study, we investigated whether [6]-gingerol prevents disruption of the TJ and cancer cell invasion in human pancreatic cancer cells.

## 2. Material and Methods

### 2.1. Cell Culture

The human pancreatic cancer cell line PANC-1 was obtained from the American Type Culture Collection (ATCC, Rockville, MD, USA). Cells were cultured in Dulbecco's modified Eagle's medium (DMEM) supplemented with 10% fetal bovine serum (FBS; Gibco-BRL, Grand Island, NY, USA) at 37°C in a humidified atmosphere containing 5% CO_2_.

### 2.2. MTT Assay

For the cell viability assay, cells were plated at 1 × 10^4^ cells/well in a 96-well plate (Nunc, Roskilde, Denmark). After incubation with 0, 5, 10, 15, or 20 *μ*M [6]-gingerol (Sigma-Aldrich, St. Louis, MO, USA) for 24 h, cell viability was determined using a 3-(4,5-dimethylthiazol-2-yl)-2,5-diphenyltetrazolium bromide (MTT) assay, which is based on the conversion of MTT to MTT-formazan by mitochondria. Cells were incubated with 1 mg/mL MTT (Chemicon, Temecula, CA, USA) in phosphate-buffered saline (PBS) for 4 h at 37°C in 5% CO_2_. Isopropanol and hydrochloric acid were then added at final concentrations of 50% and 20 mM, respectively. The optical density at 570 nm was determined using an enzyme-linked immunosorbent assay (ELISA) plate reader (MN 3663; Molecular Devices, Sunnyvale, CA, USA) with a reference wavelength of 630 nm.

### 2.3. Transepithelial Electrical Resistance (TER)

The TER is a quantitative measure explaining the barrier integrity of monolayers. The TER value was measured for transport experiments with an epithelial volt-ohm meter (World Precision Instruments, Sarasota, FL, USA). TER measurements were determined to evaluate the barrier-strengthening effect of [6]-gingerol in cells. TERs were obtained at four separate areas of each Transwell and averaged.

### 2.4. Paracellular Permeability

A PANC-1 transport study was performed to examine the effect of [6]-gingerol on paracellular permeability. Cells were seeded at a density of 6 × 10^4^ cells/cm^2^ in 12-well, 0.4 *μ*m inserts (Coaster; Corning, Corning, NY, USA) and grown in DMEM with 10% FBS for 3 days at 37°C, 95% humidity, and 5% CO_2_. Cells were washed twice with prewarmed PBS, and [^14^C] d-mannitol in PBS (0.1 *μ*Ci/mL) was then added to the apical compartment. Afterward, the basolateral sample was removed and replaced with fresh PBS at 10, 20, 30, 40, 50, and 60 min. Monolayers were continuously agitated during permeability experiments. [^14^C] d-mannitol in the samples was quantified using a LS6500TA liquid scintillation counter (Beckman Coulter, Fullerton, CA, USA). The apparent permeability coefficient, Papp (cm/s), for mannitol was determined using the following equation: Papp = (*V*_*d*_/*A*•*D*_*o*_)•(*dQ*/*dt*), where *dQ*/*dt* is the flux across the monolayer, *V*_*d*_ is the volume of the donor compartment (0.5 mL), *A* is the surface area (1 cm^2^) of the Transwell membrane, and *D*_*o*_ is the initial concentration of mannitol in the donor compartment.

### 2.5. MMP Activity

After incubation with [6]-gingerol for 24 h, cell-free culture supernatants were collected and mixed with 2× sample buffer and then subjected to 10% polyacrylamide gel electrophoresis (PAGE) with 0.1% gelatin Novex Zymogram precast gels (Invitrogen, Camarillo, CA, USA). After electrophoresis, gels were washed twice at room temperature for 30 min in 2.5% Triton X-100, subsequently washed in buffer containing 50 mM Tris-HCl, 150 mM NaCl, 5 mM CaCl_2_, 1 *μ*M ZnCl_2_, and 0.02% NaN_3_ (pH 7.5), and incubated in the same buffer at 37°C for 24 h. Gels were stained with 0.25% (w/v) Coomassie Brilliant Blue G-250 (Bio-Rad Laboratories, Hercules, CA, USA) and then destained in water solution containing methanol and acetic acid, respectively, for 1 h. The gelatinolytic activity was shown as clear bands (area of gelatin degradation) against the blue background of stained gelatin.

### 2.6. *In Vitro* Invasion Assay

To determine the effects of [6]-gingerol on PANC-1 cell invasiveness, cells were pretreated with 10 *μ*M [6]-gingerol for 6 h and plated onto the apical side of Matrigel-coated filters with 8 mm pore membranes (Corning) in serum-free medium containing either [6]-gingerol or vehicle solution. Medium containing 20% FBS was placed in the basolateral chamber to act as a chemoattractant. After 72 h, cells on the apical side were wiped off using a Q-tip. Cells on the bottom of the filter were fixed with methanol and stained with hematoxylin and eosin Y and then counted (three fields of each triplicate filter) under an inverted microscope (Nikon, Tokyo, Japan).

### 2.7. Reverse Transcription-Polymerase Chain Reaction (RT-PCR)

Total RNA was prepared using Trizol reagent (Invitrogen) according to the manufacturer's recommendations and subsequently used for RT-PCR with one step RT-PCR premix (Intron Biotechnology Co., Sungnam, Korea) according to the manufacturer's instructions. PCR was performed in a Mastercycler (Eppendorf, Hamburg, Germany) with primers indicated in Table 1. PCR conditions were as follows: 1 cycle at 94°C for 3 min; 35 cycles at 94°C for 45 s, 58°C for 45 s, and 72°C for 1 min; and 1 cycle at 72°C for 10 min. As a sample loading control and normalization between samples, PCR amplification of the housekeeping gene, glyceraldehydes 3-phosphate dehydrogenase (GAPDH), was included for each run. PCR amplification products were electrophoretically separated on a 1.5% agarose gel and visualized by ethidium bromide (EtBr; Sigma-Aldrich) staining.

### 2.8. Western Blot Analysis

Immunoblot analysis was performed to analyze protein levels. After pretreatment with the ERK inhibitor U0126 (Calbiochem, Billerica, MA, USA) for 1 h and treatment with/without [6]-gingerol for 24 h, cells were harvested, and then protein was extracted with protein lysis buffer (25 mM Tris-Cl, pH 7.5, 250 mM NaCl, 5 mM ethylendiaminetetracetic acid, 1% Nonidet P-40, protease inhibitor, and phosphatase inhibitor cocktails; Thermo Scientific, Waltham, MA, USA). Quantification of protein concentration was carried out using the Bradford method (Bio-Rad protein assay reagent), and total protein was resuspended in Laemmli sample buffer containing 5% *β*-mercaptoethanol and heated at 65°C for 10 min. Aliquots containing ~20–50 *μ*g of total cell proteins were resolved on 8–12% sodium dodecyl sulfate (SDS)-PAGE and then transferred onto nitrocellulose membranes (Amersham, Arlington Heights, IL, USA). Membranes were blocked in 5% nonfat milk (w/v) in Tris-buffered saline (TBS) containing 0.05% Tween 20 (TBST) for 1 h at room temperature, and membranes were then subjected to immunoblot analysis with the desired antibodies (Table 2). After overnight incubation at 4°C, membranes were washed in TBST and incubated with the appropriate peroxidase-conjugated secondary antibodies (Santa Cruz Biotechnology, Santa Cruz, CA, USA). Membranes were developed using chemiluminescence according to the enhanced chemiluminescence Western blotting detection reagent (Pierce, Rockford, IL, USA).

### 2.9. Electrophoretic Mobility Shift Assay (EMSA)

Nuclear proteins were extracted using the NE-PER nuclear and cytosolic extraction reagents kit (Pierce) according to the instructions. Synthetic complementary NF-*κ*B binding oligonucleotides (Promega, Madison, WI, USA) were 3-biotinylated using a biotin 3-end DNA labeling kit (Pierce) according to the manufacturer's instructions. Assays were performed using a LightShift electrophoretic mobility shift assay (EMSA) optimization kit (Pierce) according to the manufacturer's protocol.

### 2.10. Statistical Analysis

All data are presented as the means ± standard deviation (SD). Statistical analyses (Student's *t*-test and one way analysis of variance, ANOVA) were performed using GraphPad Prism 5 software (GraphPad Software, Inc., La Jolla, CA, USA). Densitometry was performed using L process V2.01 and MultiGauge V2.02 (Fuji Film, Stamford, CT, USA). A value of *P < 0.05 was considered to indicate a statistically significant difference. All results presented in the figures in this study were obtained from at least three independent experiments.

## 3. Results

### 3.1. Effect of [6]-Gingerol on Cell Viability in PANC-1 Cells

**Figure 2:**
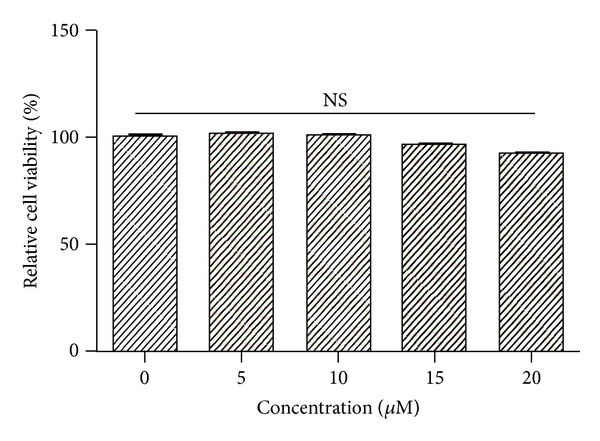
Effect of [6]-gingerol on cell viability in PANC-1 cells. Cells were seeded onto 96-well plates and grown for 1 day. Media containing the compounds were added after 24 h, and cell numbers were quantified using the MTT assay. Data represent the mean ± SD from three independent experiments. Statistical analysis (one-way ANOVA) was performed using GraphPad Prism 5. NS, no significance versus the untreated control.

The MTT assay was performed to determine the cytotoxicity of [6]-gingerol on PANC-1 cells with ~0–20 *μ*M [6]-gingerol (Figure 2). Therefore, <20 *μ*M [6]-gingerol was used for treatments in this experiment. Subsequently, [6]-gingerol did not inhibit cell growth compared to that of the control. Cytotoxicity was not observed at concentrations below 20 *μ*M [6]-gingerol.

### 3.2. [6]-Gingerol Increased Transepithelial Electrical Resistance (TER) in PANC-1 Cells

**Figure 3:**
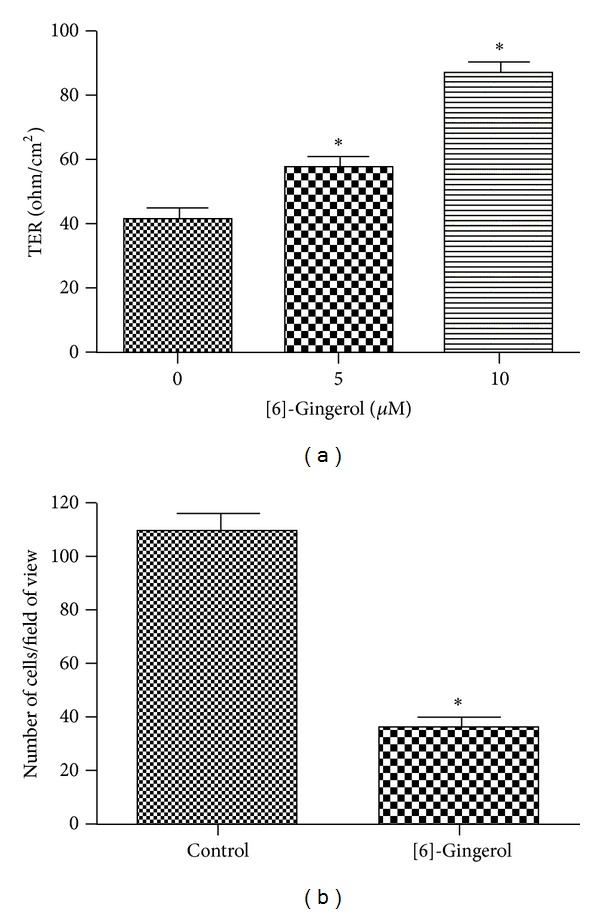
Effect of [6]-gingerol on TER value and cell invasion in PANC-1 cells. (a) Cells were plated onto 12-well polyester membrane Transwells (1 cm^2^ surface area, 0.4-mm pore size; Costar) and grown in media. The compound was added to both the apical and basolateral compartments in triplicate. TER values were measured using an epithelial volt-ohm meter. (b) Cells were grown on the apical side of a Matrigel-coated filter chamber in the presence of the compound in serum-free media. Medium including 20% FBA as a chemoattractant was placed in the basolateral chamber. After 3 days, cells were fixed and stained and then counted. Data represent the mean ± SD from three independent experiments. Statistical analyses (one-way ANOVA and Student's *t*-test) were performed using GraphPad Prism 5. *P < 0.05 versus the untreated control.

TER (a measure of TJ formation) values were measured to examine the relationship between TJ tightening and invasive activity of PANC-1 cells treated with [6]-gingerol. As shown in Figure 3(a), incubation of cells with [6]-gingerol substantially increased TER levels in a dose-dependent manner. Using a Matrigel-coated invasion assay, we next examined the question of whether [6]-gingerol decreases cell invasion activity. As shown in Figure 3(b), [6]-gingerol treatment reduced cell invasion through the Matrigel chamber. These results show that the increase in TER values upon treatment with [6]-gingerol indicates an increase in TJ formation and is associated with inhibition of cell invasion in PANC-1 cells.

### 3.3. [6]-Gingerol Reduced Paracellular Permeability in PANC-1 Cells

**Figure 4:**
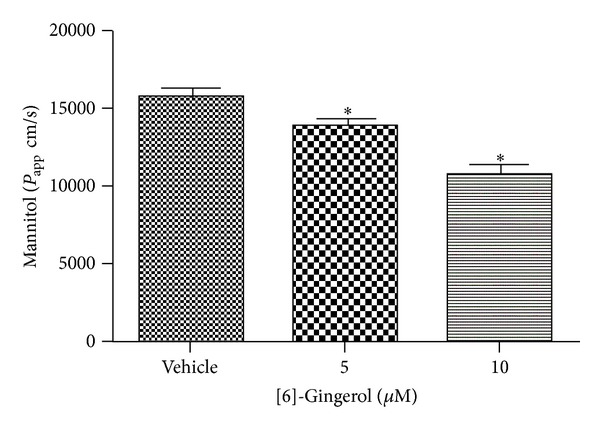
Effect of [6]-gingerol on paracellular permeability in PANC-1 cells. Cells grown on Transwells were treated for 3 days. At time 0, PBS (0.5 mL) containing [^14^C] mannitol (0.1 *µ*Ci/mL) was added to the apical compartment. The amount of mannitol transported across the monolayer was determined by counting the samples in a liquid scintillation counter (LS 6500; Beckman Coulter). Data represent the mean ± SD from three independent experiments. Statistical analysis (one-way ANOVA) was performed using GraphPad Prism 5. *P < 0.05 versus the untreated control.

To further characterize TJ changes induced by [6]-gingerol, paracellular permeability of PANC-1 monolayers was determined using the permeability marker mannitol. The d-mannitol compound is an inert carbohydrate that is transported only through this paracellular route, that is, through TJs. After 3 days of treatment with or without [6]-gingerol (10 *μ*M), the apparent permeability of mannitol (Papp mannitol) decreased by ~32% compared to that of untreated control cells (Figure 4), confirming that [6]-gingerol exhibits an enhancing effect on TJ formation in human pancreatic cancer cells.

### 3.4. [6]-Gingerol Suppressed MMP Activity in PANC-1 Cells

**Figure 5:**
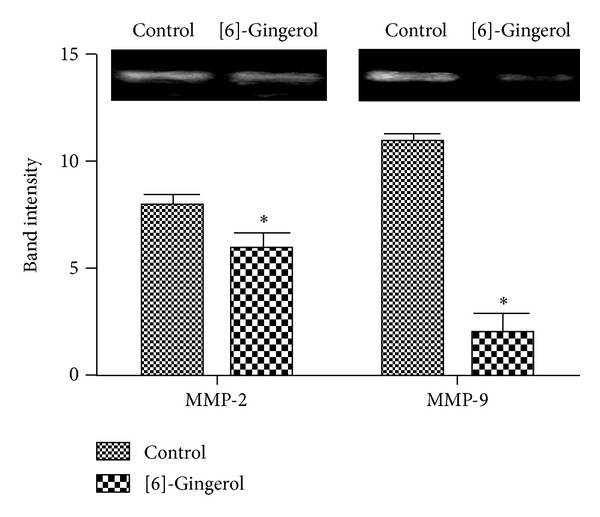
Effect of [6]-gingerol on MMP activity in PANC-1 cells. Cells were cultured in the absence or presence of the compound for 24 h. Cell-free medium was collected and MMP activity was measured by gelatin zymography. Protease activity was quantified by densitometry using L Process and MultiGauge software and normalized relative to the control and background. Data represent the mean ± SD from three independent experiments. Statistical analysis (Student's *t*-test) was performed using GraphPad Prism 5. *P < 0.05 versus the untreated control.

To clarify activation of MMPs in PANC-1 cells, zymography was conducted to assess whether [6]-gingerol affects MMP activation. Secretion of MMP-2 and MMP-9 was significantly (*P* < 0.05) inhibited by [6]-gingerol treatment compared with untreated cells (Figure 5). MMP-9 activity was suppressed more than that of MMP-2. This result suggests that [6]-gingerol inhibits the invasiveness of pancreatic cancer cells by decreasing the levels of protease, MMP-2, and MMP-9.

### 3.5. [6]-Gingerol Regulated the Expression of TJ and Invasion-Related Genes in PANC-1 Cells

**Figure 6:**
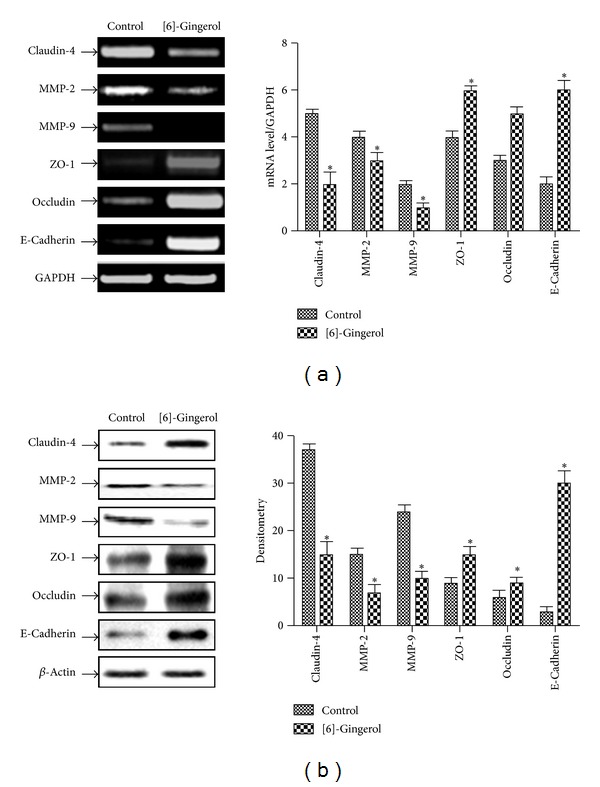
Effect of [6]-gingerol on TJ-related gene expression in PANC-1 cells. (a) Cells were treated with [6]-gingerol for 24 h. Total RNAs were extracted and reverse transcribed. cDNAs were subjected to PCR, and products were observed by 1.5% agarose gel electrophoresis and visualized by staining with EtBr. GAPDH was used as an internal control. (b) Cells cultured under the same conditions were lysed, and equal amounts of cell protein were resolved by SDS-PAGE and transferred to a nitrocellulose membrane. Western blotting was then performed with the proper antibodies and an enhanced ECL detection system. Actin was used as an internal control. Band intensities in the immunoblots were quantified by densitometry using L Process and MultiGauge software. Band intensities were normalized relative to the internal control and background, respectively. Data represent the mean ± SD from three independent experiments. Statistical analysis (Student's *t*-test) was performed using GraphPad Prism 5. *P < 0.05 versus the untreated control.

To determine whether [6]-gingerol regulates the expression of TJ and invasion-related genes, RT-PCR and Western blot analysis were conducted in PANC-1 cells with or without [6]-gingerol. As shown in Figures 6(a) and 6(b), claudin 4 and MMP-9 expression decreased in [6]-gingerol-treated cells compared to untreated cells. Inversely, the mRNA and protein levels of ZO-1, occludin, and E-cadherin increased upon treatment with [6]-gingerol. These results suggest that [6]-gingerol can restore the levels of claudin protein and that TJ may suppress metastasis and invasion.

### 3.6. [6]-Gingerol Inhibited the Invasion of PANC-1 Cells by a Decrease in NF-*κ*B/Snail Activity

**Figure 7:**
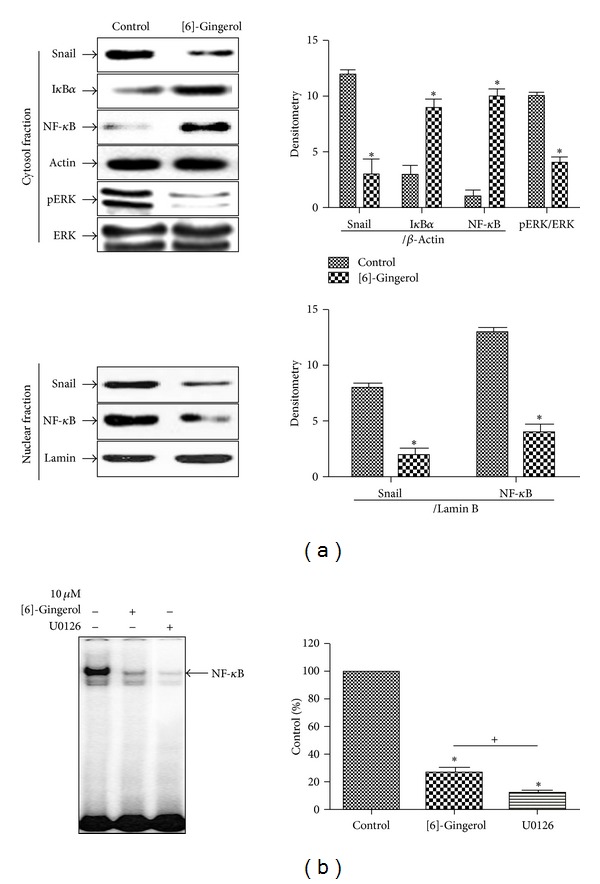
Effect of [6]-gingerol on the DNA-binding activity of NF-*κ*B in PANC-1 cells. (a) Nuclear and cytosolic extracts were prepared using the NE-PER nuclear and cytoplasmic extraction kit from PANC-1 cells treated with [6]-gingerol for 24 h. Western blotting was then performed on each fraction. Actin was used as an internal control for, the cytosolic fraction, and lamin was applied as an internal control for the nuclear fraction. (b) Cells cultured under the same conditions were pretreated for 30 min with U0126, an ERK inhibitor. Cells were then tested for the DNA-binding activity of NF-*κ*B by EMSA. Band intensities in the immunoblots were quantified by densitometry using L Process and MultiGauge software. Band intensities were normalized relative to the internal control and background. Data represent the mean ± SD from three independent experiments. Statistical analyses (Student's *t*-test and one-way ANOVA) were performed using GraphPad Prism 5. *P < 0.05 versus the untreated control. ^+^ *P* < 0.05, [6]-gingerol compared with U0126-treated cells.

As shown in Figure 7(a), protein levels of NF-*κ*B/Snail in the cytosolic fraction were significantly downregulated in [6]-gingerol-treated cells compared to those of the control. The degradation of IkB was also inhibited by [6]-gingerol. However, the nuclear translocation of NF-*κ*B and Snail significantly (*P* < 0.05) decreased in cells. Inhibition of Snail, a transcription factor, increased the expression of E-cadherin, a regulator of TJ, in cells. An inverse relationship between the expression of these genes was also observed. Activation of NF-*κ*B in the nucleus was also inhibited by [6]-gingerol treatment. MAPK is known as an upstream regulator of NF-*κ*B. Consequently, additional testing was necessary to elucidate the signaling pathway that regulates NF-*κ*B activity upon treatment with [6]-gingerol, which inhibited Snail in PANC-1 cells. Western blot analysis revealed that [6]-gingerol-treated cells suppressed ERK phosphorylation compared to that of the control (Figure 7(b)). These results demonstrate that Snail expression was inhibited by [6]-gingerol through suppression of NF-*κ*B activation via a decrease in ERK phosphorylation. As shown in Figure 7(b), treatment with U0126, an ER-specific inhibitor, confirmed the suppression of Snail and NF-*κ*B expression through inhibition of ERK phosphorylation, suggesting that inhibition of ERK by [6]-gingerol regulates NF-*κ*B activity.

## 4. Discussion

Ginger has been used in traditional Oriental medicine and contains gingerol, shogaol, paradol, zingerone, zingiberene, curcumene, and farnesene and so forth [10, 11]. Gingerols have been reported to exhibit many interesting pharmacological and physiological functions, including antipyretic, cardiotonic, chemopreventive, anti-inflammatory, and antioxidant properties[12–14].

 Metastasis is the main reason of death in patients with cancer and is a multistep process involving invasion and migration. In cancer, breakdown of the extracellular matrix and basement membrane via activation of MMPs and tissue remodeling via the loss the TJ in turn promote tumor cell migration. Here, we present a new paradigm for the prevention of PC metastasis through the restoration of TJs in PC cells by the natural compound [6]-gingerol. However, the antimetastatic effects of [6]-gingerol, a major phenolic compound derived from ginger, are unknown in PC cells. The aim of this study was to examine the effect of [6]-gingerol on PC metastasis and investigate the intracellular signaling pathways involved. 

 First, MTT assays were performed to confirm that [6]-gingerol treatment was not cytotoxic. The effects of [6]-gingerol on TER and paracellular permeability of PC cells were then investigated using the PANC-1 cell line. Our study indicated that [6]-gingerol tightened TJ formation and thus suppressed paracellular permeability compared to that of untreated cells. Soler et al. [15] demonstrated that the TER of colon carcinoma tissue was significantly lower than that of normal colon tissue and concurrently that the transepithelial paracellular permeability of colon carcinomas was higher than that in normal colon epithelial tissue, confirming the loss of TJs. Gumbiner [16] reported that the paracellular transport of ions and small solutes are regulated by TJs. Schneeberger and Lynch [17] determined that TJs are involved either directly or indirectly with TJ plaque proteins to coordinate diverse functions such as the regulation of paracellular solute permeability, cell proliferation, cell polarity, and tumor suppression. Therefore, these reports support that [6]-gingerol exhibits significant activity on two measures of metastatic potential, motility, and invasiveness in human PC cells. 

 The protein components of TJs have been identified, in particular those of the claudin family, which include transmembrane proteins and their extracellular domains. They then interact with that of other claudin proteins of adjacent cells to regulate paracellular permeability [2]. A critical regulator of TJs, ZO-1, was reduced or lost by 69% in breast cancers analyzed [18]. Claudin-1, -3, and -4 are overexpressed in colorectal tumor tissues [19]. Additionally, claudin-3 and -4 have also been shown to be overexpressed in cancers including gastric, ovarian, and pancreatic cancers [2, 20–23]. A German group reported that the ectopic expression of claudin-4 in pancreatic cancer cells reduced their invasive potential both *in vitro* and *in vivo* [2]. These reports strongly suggest that claudins may act as promising targets for antimetastatic cancer therapeutics [5]. The reports described above indicate that claudins are dysregulated in many types of cancers and that the nature of the dysregulation is highly cancer type specific. E-cadherin, an adherent junction protein and type I transmembrane glycoprotein [24], is also known to regulate TJ formation [25]. The loss of E-cadherin and Snail overexpression is correlated with tumor grade and stage [26], nodal metastasis, and tumor recurrence and predicts a poor outcome in patients with various cancers [27–29]. In this study, we showed that [6]-gingerol in PC cells inhibited TJ proteins and mRNA levels, claudin-4, ZO, and occludin. The increase in E-cadherin and decrease in Snail were also regulated by [6]-gingerol. These observations suggest that [6]-gingerol may bode well for its therapeutic use as an antimetastatic therapeutic in human pancreatic cancer. 

 MMPs are proteolytic enzymes that are highly expressed in various malignant tumors. Inhibition of MMPs could be an effective strategy to prevent tumor cell invasion and metastasis. As MMPs are important regulators of tumor progression and metastasis, they have been identified as candidate prognostic markers. Activated MMPs degrade type IV collagen (a major constituent of the basement membrane), thereby increasing cell mobility [30, 31]. Durlik and Gardian found that MMP-9 is activated only in higher tumor grades of human pancreatic cancer [32]. Dhawan et al. reported that the ectopic overexpression of claudin-1 in a colon adenocarcinoma cell line increased the activity of MMP-2 and MMP-9, which play important roles in cell invasion [33]. These reports support our findings showing that the natural compound [6]-gingerol plays inhibitory roles in metastasis and invasiveness of human pancreatic cancer cells.

 The transcription factor NF-*κ*B plays a critical role in metastasis and invasion signaling pathways [34]. Also, the transcription factor Snail translocates into the nucleus in metastatic human cancer [35]. In the current study, we found that [6]-gingerol inhibited the nuclear translocation of Snail, which is regulated by NF-*κ*B. Inhibition of Snail and MMP-9 could be the mechanism of [6]-gingerol-induced inhibition of cancer cell metastasis. We further investigated the effect of [6]-gingerol on MAPK pathways. We found that [6]-gingerol inhibited ERK phosphorylation, which was also confirmed by the U0126 inhibitor. Therefore, [6]-gingerol modulated the suppression of ERK phosphorylation, suggesting that [6]-gingerol-suppressed metastasis is associated with NF-*κ*B/Snail via the ERK pathway in PANC-1 cells.

## 5. Conclusion

In conclusion, we present data demonstrating that a natural compound, [6]-gingerol, can strengthen TJs and regulate the expressions of TJ-related proteins in human pancreatic cancer cells and that it is also a potent inhibitor of NF-*κ*B activation. Thus, our study will greatly enhance our understanding of the role of TJs and their composite proteins in human pancreatic cancer metastasis. However, further studies are needed to elucidate whether [6]-gingerol can suppress tumor metastasis and invasion *in vivo* and further potentiate chemotherapy effects.

## Figures and Tables

**Table 1:** Oligonucleotides used in RT-PCR.

Genes	Primer sequence
Claudin-4	Sense 5′-TGG ATG AAC TGC GTG GTG CAG-3′
	Antisense 5′-GAG GCG GCC CAG CCG ACG TA-3′
MMP-2	Sense 5′-GGC CCT GTC ACT CCT GAG AT-3′
	Antisense 5′-GGC ATC CAG GTT ATC GGG GA-3′
MMP-9	Sense 5′-CGG AGC ACG GAG ACG GGT AT-3′
	Antisense 5′-TCA AGG GGAAGA CGC ACA GC-3′
ZO-1	Sense 5′-GCT CCT CCC ACC TCG CAC GT-3′
	Antisense 5′-GAC CTG CTG GAG CAT AGG GCT G-3′
Occludin	Sense 5′-TCAGGGAATATCCACCTATCACTTCAG-3′
	Antisense 5′-CATCAGCAGCAGCCATGTACTCTTCAC-3′
E-cadherin	Sense 5′-GAA CAG CAC GTA CAC AGC CCT-3′
	Antisense 5′-GCA GAA GTG TCC CTG TTC CAG-3′
GAPDH	Sense 5′-CGG AGT CAA CGG ATT TGG TCG TAT-3′
	Antisense 5′-AGC CTT CTC CAT GGT GGT GAA GAC-3′

**Table 2:** List of antibodies used in Western blot.

Antibody	Company	Dilution
Claudin-4	Invitrogen	1 : 500
MMP-2	Santa Cruz Biotechnology	1 : 500
MMP-9	Santa Cruz Biotechnology	1 : 500
ZO-1	Invitrogen	1 : 1000
Occludin	Invitrogen	1 : 1000
E-cadherin	Invitrogen	1 : 1000
Snail	Abcam	1 : 1000
NF-*κ*B	Santa Cruz Biotechnology	1 : 1000
pERK	Cell Signaling	1 : 1000
ERK	Cell Signaling	1 : 1000
*β*-actin	Santa Cruz Biotechnology	1 : 1000
Lamin B	Santa Cruz Biotechnology	1 : 1000
